# Supplementary material for: Reconciling Mining with the Conservation of Cave Biodiversity: A Quantitative Baseline to Help Establish Conservation Priorities
Source: PLoS One. 2016 Dec 20;11(12):e0168348. doi: 10.1371/journal.pone.0168348 (PMC5173368; doi:10.1371/journal.pone.0168348)
Supplement: S1 Dataset — (ZIP) [file pone.0168348.s002.zip › Taxa/Serra Norte/SN_2013/N4E/Lista N4E-63.pdf]

CAVIDADE N4E-0063

Classe	Ordem	Fam/Outros	Gên/Outros	Espécie	S-U	U-S	Zon.
Arachnida	Acari	(Oribatida)		sp.8		X	E
Arachnida	Acari	Anystidae	*Erythracarus*	sp.1	X		E
Arachnida	Acari	Ixodidae	*Amblyomma*	sp.1		X	E
Arachnida	Acari	Podocinidae	*Podocinum*	sp.1	X		E
Arachnida	Acari	Prostigmata		sp.49	X		E
Arachnida	Amblypygi	Charinidae	*Charinus*	sp.1		1	E
Arachnida	Araneae	Araneidae	*Alpaida*	aff. *negro*		X	E
Arachnida	Araneae	Araneidae	*Alpaida*	*antonio*		X	E
Arachnida	Araneae	Corinnidae		jovem	1		E
Arachnida	Araneae	Ctenidae		jovem	1		E
Arachnida	Araneae	Filistatidae		sp.1	X		E
Arachnida	Araneae	Ochyroceratidae		jovem		X	E
Arachnida	Araneae	Palpimanidae		jovem		X	E
Arachnida	Araneae	Pholcidae	*Leptopholcus*	sp.1		X	E
Arachnida	Araneae	Pholcidae	*Mesabolivar*	*aurantiacus*		X	E
Arachnida	Araneae	Pholcidae	*Mesabolivar*	sp.1	X	X	E
Arachnida	Araneae	Scytodidae	*Scytodes*	*eleonorae*		1	E
Arachnida	Araneae	Segestriidae		jovem	X		E
Arachnida	Araneae	Theridiidae	*Dipoena*	sp.4	X		E
Arachnida	Araneae	Theridiosomatidae	*Plato*	sp.1		X	E
Arachnida	Pseudoscorpiones	Olpiidae		sp.1		X	E
Chilopoda	Scolopendromorpha	Scolopocryptopidae		jovem		1	E
Diplopoda	Polydesmida	Fuhrmannodesmidae		jovem	X		E
Diplopoda	Polydesmida	Paradoxosomatidae		sp.1		1	E
Diplopoda	Polyxenida	Polyxenidae		jovem	X		E
Entognatha	Collembola	Entomobryidae		sp.1	X		E
Entognatha	Collembola	Paronellidae		sp.1	X	X	E
Entognatha	Collembola	Paronellidae		sp.6	X		E
Insecta	Blattodea			jovem	1	1	E
Insecta	Coleoptera	Chrysomelidae		sp.9	X		E
Insecta	Diptera	Asilidae		sp.	X		E
Insecta	Diptera	Cecidomyiidae	Cecidomyiinae	sp.	X	X	E
Insecta	Diptera	Culicidae	*Culex*	sp.	X		E
Insecta	Diptera	Dolichopodidae		sp.	X		E
Insecta	Diptera	Drosophilidae		sp.	X		E
Insecta	Diptera	Psychodidae	Phlebotominae	sp.		X	E
Insecta	Diptera	Tipulidae	Tipulinae	sp.	X	X	E
Insecta	Heteroptera	Pentatomoidea		sp.	X		E
Insecta	Homoptera	Cixiidae		jovem		X	E
Insecta	Hymenoptera	Formicidae	*Camponotus*	sp.1	X		E
Insecta	Hymenoptera	Formicidae	*Camponotus*	sp.2	X	X	E
Insecta	Hymenoptera	Formicidae	*Eciton*	sp.1		X	E
Insecta	Hymenoptera	Formicidae	*Eurhopalothrix*	sp.1		X	E
Insecta	Hymenoptera	Formicidae	*Gnamptogenys*	sp.1		X	E
Insecta	Hymenoptera	Formicidae	Myrmicinae	sp.1	X		E
Insecta	Hymenoptera	Formicidae	*Pachycondyla*	sp.1	X		E
Insecta	Isoptera	Termitidae	*Nasutitermes*	sp.	X	X	E
Insecta	Lepidoptera	Noctuoidea		sp.1		1	E
Insecta	Lepidoptera	Noctuoidea		sp.5	1	5	E

Insecta	Lepidoptera	Tineoidea		sp.3	X		E
Insecta	Orthoptera	Phalangopsidae	*Paraclodes*	sp.	8	4	E
Insecta	Psocoptera	Manicapsocidae	*Nothoentomum*	sp.1	X	X	E
Insecta	Thysanura	Nicoletiidae		sp.1		X	E
Malacostraca	Decapoda	Pseudothelphusidae		sp.1	1		E
Amphibia	Anura	Strabomantidae	*Pristimantis*	cf. *fenestratus*	1	1	E
Mammalia	Chiroptera	Emballonuridae	*Peropteryx*	*kappleri*	1	1	E
